# The Werner Syndrome Helicase/Exonuclease Processes Mobile D-Loops through Branch Migration and Degradation

**DOI:** 10.1371/journal.pone.0004825

**Published:** 2009-03-13

**Authors:** Patricia L. Opresko, Gregory Sowd, Hong Wang

**Affiliations:** 1 Department of Environmental and Occupational Health, University of Pittsburgh Graduate School of Public Health, Pittsburgh, Pennsylvania, United States of America; 2 Department of Pharmacology and Chemical Biology, University of Pittsburgh School of Medicine, Pittsburgh, Pennsylvania, United States of America; 3 The University of Pittsburgh Cancer Institute, Hillman Cancer Center, Pittsburgh, Pennsylvania, United States of America; Ordway Research Institute, United States of America

## Abstract

RecQ DNA helicases are critical for preserving genome integrity. Of the five RecQ family members identified in humans, only the Werner syndrome protein (WRN) possesses exonuclease activity. Loss of WRN causes the progeroid disorder Werner syndrome which is marked by cancer predisposition. Cellular evidence indicates that WRN disrupts potentially deleterious intermediates in homologous recombination (HR) that arise in genomic and telomeric regions during DNA replication and repair. Precisely how the WRN biochemical activities process these structures is unknown, especially since the DNA unwinding activity is poorly processive. We generated biologically relevant mobile D-loops which mimic the initial DNA strand invasion step in HR to investigate whether WRN biochemical activities can disrupt this joint molecule. We show that WRN helicase alone can promote branch migration through an 84 base pair duplex region to completely displace the invading strand from the D-loop. However, substrate processing is altered in the presence of the WRN exonuclease activity which degrades the invading strand both prior to and after release from the D-loop. Furthermore, telomeric D-loops are more refractory to disruption by WRN, which has implications for tighter regulation of D-loop processing at telomeres. Finally, we show that WRN can recognize and initiate branch migration from both the 5′ and 3′ ends of the invading strand in the D-loops. These findings led us to propose a novel model for WRN D-loop disruption. Our biochemical results offer an explanation for the cellular studies that indicate both WRN activities function in processing HR intermediates.

## Introduction

Werner syndrome (WS) is an autosomal recessive disorder marked by the premature onset of numerous features associated with aging and a predisposition to mesenchymal cancers [1]. WS is caused by loss of the DNA repair protein WRN which is a member of the RecQ family of DNA helicases [2]. *E. coli* and *S. cerevisiase* each have a single family member, whereas five members exist in humans: RECQ1, RECQ4, RECQ5, BLM and WRN [3], [4]. Mutations in the human RecQ helicases BLM and RECQ4 also give rise to the cancer predisposition disorders Bloom syndrome (BS) and Rothmund-Thomson syndrome (RTS), respectively [5]. Despite the common feature of genomic instability, these disorders are clinically distinct from WS. While there are no known human disorders caused by defects in RECQ1 or RECQ5, cellular and transgenic mouse studies indicate that loss of these proteins also leads to genomic instability [5]. Thus, RecQ helicases have been classified as “caretakers” of the genome [3].

RecQ helicases have critical roles in regulating homologous recombination (HR) pathways. HR functions in repair of DNA double strand breaks, restoration of collapsed replication forks, and the alternative lengthening of telomeres (ALT) pathway [6]–[9]. Inappropriate HR can lead to loss of heterozygosity due to DNA strand crossovers, chromosome translocations, telomere loss, and tangled DNA intermediates that are potentially toxic if left unresolved [6]. In HR broken DNA ends are processed to generate single stranded tails that are coated by Rad51, which catalyzes invasion of the ssDNA tail into homologous duplex sequence and promotes base pairing between the ssDNA tail and one strand of the duplex [10]. The result is a joint molecule termed a displacement loop (D-loop). If the invading ssDNA terminates in a 3′OH it primes DNA polymerase extension and copying of the homologous sequence [8], [11]. Dissociation of the D-loop can lead to re-annealing of the displaced strand to the ssDNA tail of the other broken end in the synthesis-dependent strand annealing (SDSA) pathway [12]. Alternatively, the tail of the other broken end may also be captured leading to the formation of a double Holliday junction (HJ), which is processed into strand crossover or non-crossover products [13]. RecQ helicases regulate HR by preventing inappropriate events through displacing Rad51 (BLM, RECQ1, RECQ5) [14]–[16] and by dissociating recombination intermediates including various HJ and D-loop constructs [16]–[20]. Unwinding of duplex DNA is catalyzed by RecQ helicases with 3′ to 5′ polarity and requires nucleotide hydrolysis.

WRN is unique among the human RecQ helicases in that the protein also has 3′ to 5′ exonuclease activity [21], and both helicase and exonuclease activities are implicated in the dissociation of HR intermediates. WS cells exhibit defects in resolving HR intermediates that form in response to stalled replication forks, whether induced by blocking lesions or by depleted dNTP pools [22], [23]. The HR defect can be rescued by inhibiting Rad51 [24], and thus the formation of recombination intermediates, but is not rescued by expressing WRN mutants that lack either helicase or exonuclease activities [25]. WRN deficient cells are also prone to abnormal HR at telomeric ends, and exhibit increased telomere loss, telomeric sister chromatid exchanges and spontaneous extra-chromosomal telomeric circles [26]–[29]. Telomeres contain a 3′ single strand (ssDNA) tail that is ∼50 to 150 nt long *in vivo* [30] and forms protective intra-telomeric D-loops that stabilize the large t-loop [31]. Aberrant HR involving the natural D-loop/t-loop or inappropriate strand invasion into telomeric sequences in other chromatids or chromosomes can lead to telomere loss and abnormal telomere structures [32], [33]. RecQ helicases are also implicated in the resolution step of the recombination-based ALT pathway to lengthen telomeres [34], [35].

WRN acts on many of the same substrates as other human RecQ helicases, but has the potential to process these substrates differently due to the exonuclease activity. Precisely how both WRN biochemical activities contribute to processing HR intermediates is not clear. We and others observed previously that oligomeric non-mobile D-loops are substrates for both the WRN helicase and exonuclease activities, but these constructs could not test for branch migration activity [36], [37]. WRN helicase activity is poorly processive and is unable to completely unwind a 33-bp oligomeric telomeric D-loop in the absence of accessory proteins POT1 or RPA, or assistance from the WRN exonuclease activity [37], [38]. Exonucleolytic degradation shortens the duplex length to be unwound by digesting the invading strand in a 3′ to 5′ direction [37]. Here we extended our previous studies by constructing more biologically relevant mobile D-loops. They have a much longer invading strand that pairs with one strand in a plasmid to form an 84 bp duplex, and can be branch migrated. Based on evidence that WRN regulates recombination at telomeres [39], we also constructed telomeric D-loops for analysis.

In this study we identified mobile plasmid-based D-loops as novel substrates for both WRN helicase driven branch migration and exonuclease activities, and found that telomeric D-loops were disrupted less efficiently than non-telomeric D-loops. In contrast to oligomeric constructs, we show that WRN helicase alone can completely dissociate the 84 bp duplex to release the invading strand from the plasmid D-loop. However, the WRN exonuclease activity alters processing by degrading the long invading strand both prior to and after release from the plasmid D-loop. WRN disrupts plasmid D-loops with a protruding 3′ or 5′ single stranded tail, but a protruding tail is not required for either branch migration or exonuclease activities. Furthermore, we demonstrate that WRN recognizes both the 5′ and 3′ ends of the D-loop duplex, which led us to propose a novel model for WRN loading and processing of biologically relevant D-loop recombination intermediates.

## Results

### WRN disrupts mobile plasmid D-loops

**Figure 1:**
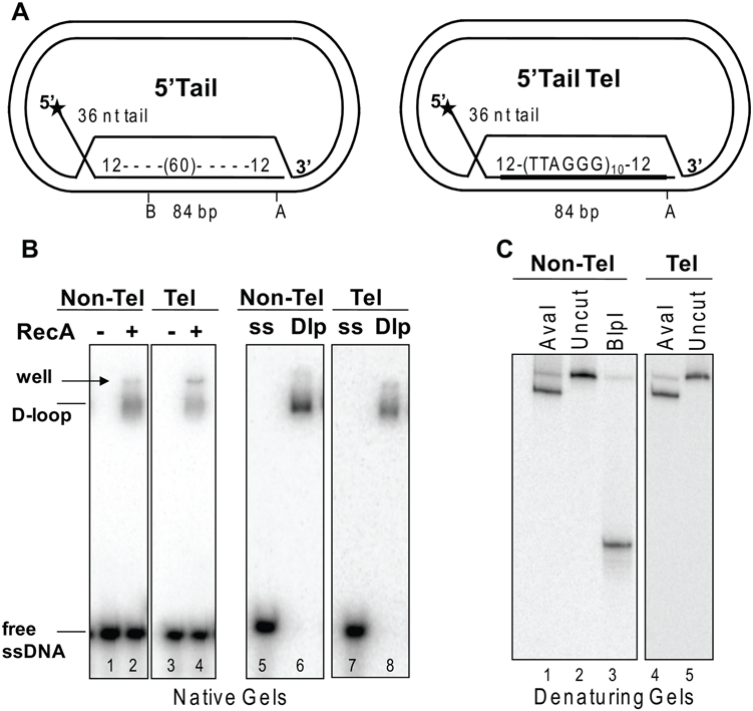
Construction of mobile plasmid D-loops. A. Schematic of 5′ Tail non-telomeric and telomeric (Tel) plasmid D-loops. The star denotes the 5′ end radiolabel. The invading strand of the 5′Tail Tel D-loop base pairs with the plasmid to form an 84 bp duplex with ten (TTAGGG) repeats flanked by 12 bp of unique sequence. The telomeric repeats are scrambled in the non-telomeric D-loop. Restriction enzyme sites are indicated as A, AvaI; B, BlpI. B. RecA generated plamid D-loops are stable. Plasmids (300 µM nucleotides) were mixed with the 5′end-labeled complementary oligonucleotide (3.6 µM nucleotides) and RecA protein (4 µM) (lanes 2 and 4). D-loops (Dlp) were then purified from unincorporated oligonucleotide (ss) (lanes 6 and 8). Reactions were run on a 4–20% polyacrylamide native gel. C. The D-loop invading strand is fully base paired with the plasmid strand. Reactions (20 µl) contained 50 pM purified 5′Tail non-telomeric (Non-Tel) or telomeric (Tel) D-loops and 10 units of the indicated restriction enzyme. Products were run on a 14% polyacrylamide denaturing gel.

To generate more biologically relevant D-loops we used RecA to catalyze the invasion of a 120-mer oligonucleotide into negatively supercoiled plasmids. The resulting D-loop contained a 5′ 36 nt ssDNA tail protruding from an 84 bp duplex in which the oligonucleotide base paired with the complementary strand of the plasmid (Fig. 1A–B). The strand invasion duplex sequence for the 5′Tail Telomeric (Tel) D-loop consisted of (TTAGGG)_10_ flanked by 12 bp of unique sequence to ensure proper alignment of the repeats. The telomeric repeat sequence was scrambled for the non-telomeric 5′Tail D-loop. Both D-loops were stable since no spontaneously released oligonucleotide was detected after purification (Fig. 1B, lanes 6 and 8). The duplex region of the non-telomeric D-loop was susceptible to cleavage by restriction enzymes indicating the invading strand was fully based paired with the plasmid strand (Fig. 1C). Finally, the 3′ 12 bp flanking sequence in the duplex of both D-loops contained an AvaI restriction site that was cleaved to near completion confirming proper based pairing at the 3′end of the invading strand (Fig. 1C). These data demonstrate that the mobile D-loops are stable and contain an invading strand that is completely base paired with the complementary plasmid strand.

**Figure 2:**
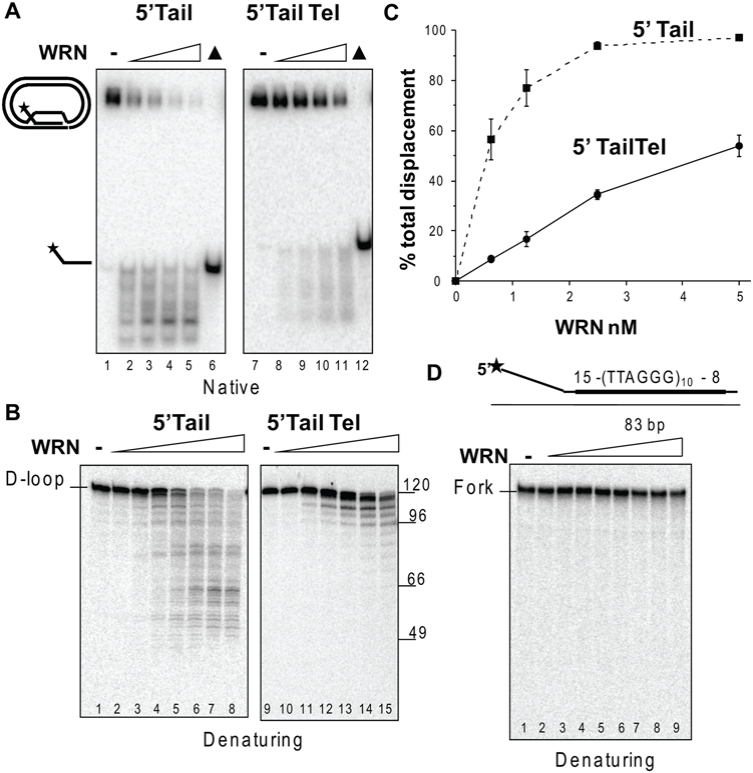
WRN displaces and digests the invading strand of plasmid D-loops. A. Reactions contained 50 pM of either the 5′Tail non-telomeric (lane 1–6) or the telomeric (Tel) (lanes 7–12) plasmid D-loops. The substrate was incubated with WRN concentrations increasing in doubling steps from 0.62 to 5 nM (lanes 2–5 or 8–11, respectively) for 15 min under standard reaction conditions and run on a 4–20% native polyacrylamide gel. ▴, heat denatured substrate. B. The 5′ Tail non-telomeric (lanes 1–8) or telomeric (lanes 9–15) were incubated with WRN concentrations increasing in doubling steps from 0.078 to 5 nM (lanes 2–8) and 0.15 to 5 nM (lanes 10–15) and run on a 14% denaturing polyacrylamide gel. Numbers represent oligonucleotide size markers. C. The percent total D-loop displacement from reactions in panel A were calculated as described in Materials and Methods and plotted against WRN concentration. WRN and 5′Tail plasmid D-loop, ▪ and dotted line; WRN and 5′Tail Tel plasmid D-loop, • and solid line. Values represent the mean and standard deviation (SD) from at least three independent experiments. D. WRN exonuclease is inactive on an 83 bp forked duplex. Reactions contained 0.25 nM of the 83-bp forked duplex containing ten telomeric repeats (thick black line) with 15 and 8 bp of flanking sequence. The substrate was incubated with WRN concentrations increasing in doubling steps from 0.19 to 25 nM for 15 min under standard reaction conditions. Reactions were run on a 14% denaturing gel.

To examine WRN catalytic activities on the plasmid D-loops, the substrates were incubated with increasing WRN concentrations. The reactions were analyzed on a native gel to display D-loop disruption and on a denaturing gel to better visualize the products of the 3′ to 5′ exonuclease activity. WRN protein released a mixture of full length and shortened strands due to the combined action of strand displacement activity and the exonuclease digesting from the 3′OH of the invading strand (Fig. 2A–B). The telomeric sequence was more refractory to displacement and digestion (Fig. 2A–C). Approximately 10-fold more WRN was required to disrupt 50% of the telomeric 5′Tail Tel D-loop (5 nM WRN) compared to the non-telomeric 5′Tail D-loop (0.5 nM WRN). The exonuclease progressed further through the non-telomeric sequence with a maximum of ∼73 nt digested (47 nt product), compared to the telomeric D-loop with a maximum of ∼55 nt digested (66 nt product) (Fig. 2B). The distinct pattern of digestion products for the telomeric 5′Tail Tel D-loop corresponds to termination at the G run of each repeat (Fig. 2B, lanes 9–15) as observed previously for oligomeric forks and D-loops [37], [40]. To examine the mechanism of WRN D-loop displacement, we constructed a forked duplex of similar size and sequence as the telomeric D-loop. Consistent with previous reports [41], an 83 bp duplex is too long for WRN helicase activity to efficiently unwind (Supplemental Fig. S1). This result, along with the finding that WRN helicase cannot completely unwind a 33-bp non-mobile D-loop [37], supports the conclusion that WRN disrupts the 84-bp mobile D-loops through branch migration rather than simply strand separation. Furthermore, even though WRN can digest at the blunt end of a shorter fork (34-bp) [42], WRN exonuclease fails to digest the 83-bp forked duplex (Fig. 2D). Therefore, the plasmid D-loops are preferred substrates for displacement and exonucleolytic attack by WRN protein. In addition, both non-telomeric and telomeric plasmid D-loops are substrates for WRN branch migration and exonucleolytic attack, although the telomeric sequence is more refractory to processing.

### WRN can branch migrate the full 84 bp duplex of the plasmid D-loop

**Figure 3:**
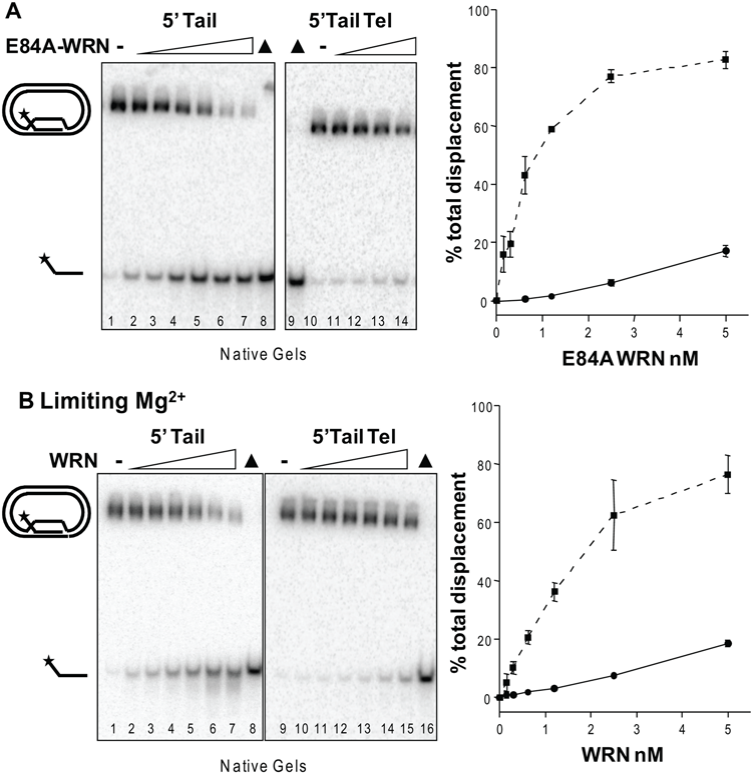
WRN can branch migrate through the full 84 bp duplex to displace the invading strand. Reactions contained 50 pM of either the 5′Tail non-telomeric (lanes 1–8) or the telomeric (Tel) (lanes 9–14 (A.) or 9–16 (B.)) plasmid D-loops. Reactions were run on 4–20% native polyacrylamide gels and visualized by phosphorimager analysis. ▴, heat denatured substrate. A. Branch migration after exonuclease inactivation with a WRN mutant. The substrate was incubated with E84A-WRN concentrations increasing in doubling steps from 0.15 to 5 nM (lanes 2–7) or from 0.62 to 5 nM (lanes 11–14) for 15 min under standard conditions. B. Branch migration after exonuclease inhibition with limiting Mg^2+^. The substrate was incubated with wild type WRN concentrations increasing in doubling steps from 0.15 to 5 nM (lanes 2–7, and lanes 10–15) for 15 min in reactions buffer containing 1 mM MgCl_2_ and 2 mM ATP. The percent total displacement was plotted against E84A-WRN (A.) or wild type WRN (B.) concentration. E84A-WRN or WRN and 5′Tail D-loop, ▪ and dotted line; E84A-WRN or WRN and 5′Tail Tel D-loop, • and solid line. Values are the mean and standard deviation (SD) from two-three independent experiments.

To confirm that WRN helicase branch migration activity alone can disrupt the full 84 bp duplex in the plasmid D-loops, we inactivated the WRN exonuclease activity. For this we incubated the 5′Tail non-telomeric and telomeric D-loops with an exonuclease-dead variant of WRN (E84A). Branch migration through the full 84-bp duplex was observed for both D-loops, and was detected at as low as a 3-fold molar excess of E84A-WRN (0.15 nM) over substrate (0.05 nM) for the non-telomeric 5′Tail D-loop (Fig. 3A, lane 2). However, the total percent of D-loops disrupted for the E84A-WRN mutant was decreased compared to wild type WRN (compare Figs. 2A and 3A). In order to examine branch migration activity of the wild type WRN protein preparation in the absence of digestion, we suppressed the WRN exonuclease activity by decreasing the free Mg^2+^ concentration. Since the WRN exonuclease acts by a two-metal ion dependent mechanism, free divalent cations are required to bind the exonuclease active site [43]–[45]. While WRN helicase remains active at Mg^2+^∶ATP ratios near 1 [43](data not shown), we found that WRN exonuclease activity is highly dependent on the free Mg^2+^ concentration. The incubation of WRN and the 5′Tail D-loops in reactions with a molar excess of ATP over Mg^2+^ (2∶1) resulted in the loss of shortened products in favor of displaced full length strands (Fig. 3B). Decreasing the Mg^2+^ concentration inhibited the WRN exonuclease activity and reduced the percent of total disrupted D-loops (compare Figs. 2A and 3B). In summary, WRN protein can branch migration through the full 84-bp duplex of the plasmid D-loops, but the percent of D-loop disruption is decreased in the absence of exonuclease activity.

### WRN exonuclease activity digests the invading strand prior to and after release from the D-loop

**Figure 4:**
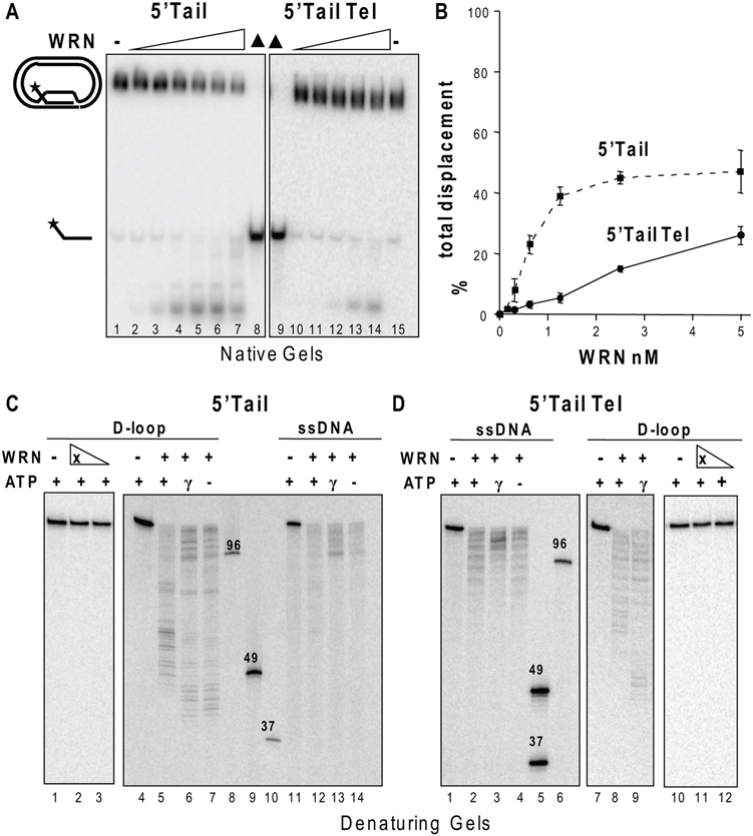
WRN exonuclease digests the long invading strands both prior to and after release from the plasmid D-loops. A. Reactions contained 50 pM of either the 5′Tail non-telomeric (lane 1–8) or the telomeric (Tel) (lanes 9–15) plasmid D-loops. The substrate was incubated with WRN concentrations increasing by doubling steps from 0.15 to 5 nM (lanes 2–7) or from 0.31 to 5 nM (lanes 10–14) for 15 min under standard reaction conditions except ATP was replaced with ATPγS. Reactions were run on 4–20% native polyacrylamide gels and visualized by phosphorimager analysis. ▴, heat denatured substrate. B. The percent total displacement was calculated as described in Materials and Methods and plotted against WRN concentration. WRN and 5′Tail D-loop, ▪ and dotted line; WRN and 5′Tail Tel D-loop, • and solid line. Values are the mean and standard deviation (SD) from two independent experiments. C. and D. WRN exonuclease on intact and released invading strands. Reactions contained 50 pM of either the non-telomeric (C.) 5′Tail D-loop (lanes 1–7) or 120-mer oligonucleotide (lanes 11–14) or the telomeric (D.) 5′Tail Tel D-loop (lanes 7–12) or 120-mer oligonucleotide (lanes 1–4). The substrates were incubated with WRN (5 nM) or E84A-WRN mutant (X; 5 and 2.5 nM) under standard conditions for 15 min except that either 2 mM ATP, 2 mM ATPγS (γ) or no ATP (-) was added as indicated. Reactions were run on 14% polyacrylamide denaturing gels. Oligonucleotide size markers were loaded for reference.

Next we asked whether the WRN exonuclease digested the invading strand in the context of the D-loop and/or after displacement. The long length of the invading strand (120 nt) in the plasmid D-loops makes it a potential substrate for WRN exonucleolytic attack after release from the D-loop, in contrast to the previously tested 33-bp oligomeric D-loops [37]. WRN exonuclease is inefficient on short ssDNA strands (<40 nt), but extensively digests long 80-mer oligonucleotides due to increased affinity for long ssDNA molecules [46]. To test whether WRN can digest the invading strand in the context of the D-loop, we inhibited branch migration activity by using the analog ATPγS that is not efficiently hydrolyzed by WRN [47]. Branch migration of mobile D-loops by RecQ helicases RECQ1 and BLM require the energy from ATP hydrolysis [16], [19], [48]. Disruption of the 5′Tail D-loops occurred even in the absence of WRN branch migration activity but was reduced approximately 2.5-fold for both the D-loops within the linear range of the titration curve (Fig. 4A–B). However, we observed a loss of the full length products and the appearance of very short ssDNA products that resulted from extensive digestion of the duplex to thermally unstable lengths (Fig. 4A). Similar results were obtained in the absence of ATP or the analog (data not shown). Visualization of the exonuclease products on a denaturing gel clearly shows the extent D-loop digestion progressed further when ATP hydrolysis is inhibited (ATPγS and no ATP) (Fig. 4C, compare lanes 5 to 6 and 7, Fig. 4D compare lanes 8 to 9) for some molecules. The exonuclease-dead WRN mutant (E84A) yielded no detectable digestion products after incubation with either D-loop (Fig. 4C lanes 1–3, Fig. 4D lanes 10–12).

Next we asked if WRN can digest the long invading strands after branch migration released them from the D-loop, by incubating WRN with the 120 nt oligomers used to construct the 5′Tail D-loops (Table 1). WRN digestion of the long oligomers was apparent (Figs. 4C lanes 11–14, and 4D lanes 1–4), dose dependent and eliminated upon mutation of the WRN exonuclease domain (Supplemental Fig. S2). The extent of WRN digestion of the long ssDNA molecules was not increased in the absence of ATP hydrolysis (Fig. 4C, lanes 12–14 and Fig. 4D, lanes 2–4), in contrast to digestion of the 5′Tail D-loops. In summary, WRN exonucleolytic attack of the D-loop invading strand does not require branch migration activity and occurs prior to and after release from the D-loop.

**Table 1:** Oligonucleotides used in substrate preparations.

Name	Sequence (5′ to 3′)
Fork-C	CAA CGC CGT ACG TCG GTT GCT ATG GCC TCG AGA CCC TAA CCC TAA CCC TAA CCC TAA CCC TAA CCC TAA CCC TAA CCC TAA CCC TAA CCC TAA ACG CGC AGC CTG GTC **TTT TTT TTT TTT TTT TT**
Fork-G	**TT CAC GCG TCT GCG TTC** GAC CAG GCT GCG CGT TTA GGG TTA GGG TTA GGG TTA GGG TTA GGG TTA GGG TTA GGG TTA GGG TTA GGG TTA GGG TCT GCA GGC CAT AGC AAC CGA GCT ACG GCG TTG
5′Tail Tel	**AAT TCT CAT TTT ACT TAC CTG ACG CTA TTA GCA GTG** CAG GCT GCG CGT TTA GGG TTA GGG TTA GGG TTA GGG TTA GGG TTA GGG TTA GGG TTA GGG TTA GGG TTA GGG TCT CGA GGC CAT
5′Tail	**AAT TCT CAT TTT ACT TAC CTG ACG CTA TTA GCA GTG** CAG GCT GCG CGT ATC GGT ATT GGC TTA GCA CTG GCA ATC GGT CTT GCA CTG GCT ATT GGC TTA GGT ATC GCA TCT CGA GGC CAT
3′ Tail	CAG GCT GCG CGT ATC GGT ATT GGC TTA GCA CTG GCA ATC GGT CTT GCA CTG GCT ATT GGC TTA GGT ATC GCA TCT CGA GGC CAT **AAT TCT CAT TTT ACT TAC CTG ACG CTA TTA GCA GTG**
No Tail	CAG GCT GCG CGT ATC GGT ATT GGC TTA GCA CTG GCA ATC GGT CTT GCA CTG GCT ATT GGC TTA GGT ATC GCA TCT CGA GGC CAT
5B3B Tails	**TCT CAT TTT ACT TAC GTC** CAG GCT GCG CGT ATC GGT ATT GGC TTA GCA CTG GCA ATC GGT CTT GCA TCT CGA GGC CAT **ATT TCT CAT TTT ACT TAC**
5B Tails	**TCT CAT TTT ACT TAC GTC** CAG GCT GCG CGT ATC GGT ATT GGC TTA GCA CTG GCA ATC GGT CTT GCA TCT CGA GGC CAT **ATT TCT CAT TTT ACT TAC**
3B Tails	**TCT CAT TTT ACT TAC GTC** CAG GCT GCG CGT ATC GGT ATT GGC TTA GCA CTG GCA ATC GGT CTT GCA TCT CGA GGC CAT **ATT TCT CAT TTT ACT TAC**
5B Tail, no 3′Tail	**TCT CAT TTT ACT TAC GTC** CAG GCT GCG CGT ATC GGT ATT GGC TTA GCA CTG GCA ATC GGT CTT GCA TCT CGA GGC CAT

Bolded letters indicate ssDNA regions in the final construct.

Strikethrough indicates that the nucleotides were removed in the final construct.

Underlined letter indicates a biotinylated nucleotide.

### Structural requirements of WRN D-loop branch migration and exonuclease activities

**Figure 5:**
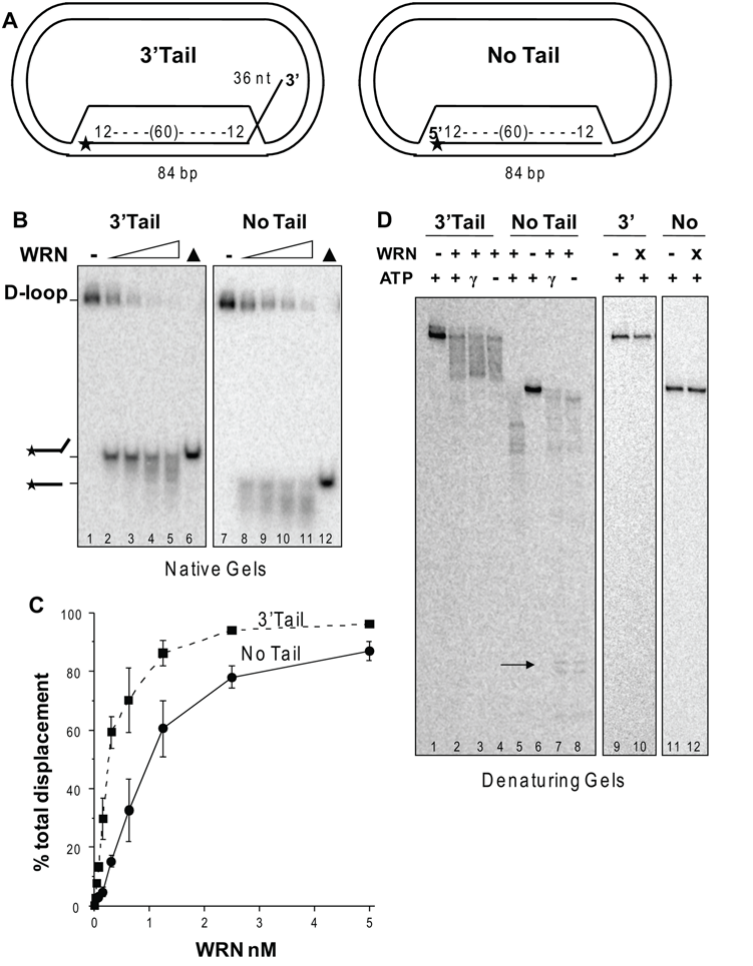
Structural requirements for WRN activity on plasmid D-loops. A. Schematic of 3′Tail and No Tail D-loops. The star denotes the 5′ end radiolabel. B. Reactions contained 50 pM of either the 3′Tail (lane 1–6) or the No Tail (lanes 7–12) plasmid D-loops. The substrate was incubated with WRN concentrations increasing by doubling steps from 0.62 to 5 nM (lanes 2–5 and 8–11) for 15 min under standard reaction conditions. Reactions were run on 4–20% polyacrylamide native gels and visualized by phosphorimager analysis. ▴, heat denatured substrate. C. The percent total displacement was calculated as described in Materials and Methods and plotted against WRN concentration. WRN and 3′Tail D-loop, ▪ and dotted line; WRN and No Tail D-loop, • and solid line. Values are the mean and standard deviation (SD) from at least three independent experiments. D. Analysis of WRN exonuclease activity. Reactions contained 50 pM of either the 3′Tail (lanes 1–4 and 9–10) or the No Tail (lanes 5–8 and 11–12) D-loops. The substrates were incubated with 5 nM WRN or E84A-WRN mutant (X) under standard conditions for 15 min except that either 2 mM ATP, 2 mM ATPγS (γ) or no ATP (-) was added as indicated. Reactions were run on 14% polyacrylamide denaturing gels. Arrow points to products representing digestion almost to the end of the 84-bp duplex.

To determine the structural requirements for WRN catalytic activities on the plasmid D-loops, we constructed D-loops with either a protruding 3′ ssDNA tail or no protruding tail (Fig. 5A). WRN disrupted about 50% of the D-loops at ∼0.25 nM and 1 nM protein for the 3′Tail D-loop and No Tail D-loop, respectively, and released a mix of full length and shortened strands (Fig. 5A–C). Importantly, disruption of the 3′Tail D-loop (7.6%) was detected at near equal molar WRN (0.04 nM) and substrate (0.05 nM) concentrations (Fig. 5C). No digestion of the D-loops is detected when the WRN exonuclease domain is mutated (E84A-WRN) (Fig. 5D, lanes 9–12). To ensure that WRN can digest the strands in the context of the D-loop we also examined processing in the absence of branch migration (ATPγS and no ATP). The extent of digestion of the No Tail D-loop invading strand was similar to the 5′Tail D-loop both in the presence of branch migration activity (Fig. 5D lane 5 compare to Fig. 4C lane 5), and in the absence of branch migration activity in which digestion progressed almost to the end of the duplex (Fig. 5D lanes 7–8 compare to Fig. 4C lanes 6–7). However, the 3′Tail D-loops differ in that the exonuclease initiates digestion on ssDNA rather than on duplex DNA. WRN digestion of the 3′Tail D-loop in the absence of branch migration (Fig. 5D, lanes 3–4) confirms that WRN can degrade a 36 nt 3′ssDNA tail protruding from the plasmid D-loop. We did not detect any appreciable digestion into the duplex for the 3′Tail D-loop (Fig. 5D, lanes 3–4), which is expected since WRN needs to degrade the full 36 nt tail prior to encountering the duplex. Consistent with a failure to shorten the duplex region, no disruption of the 3′Tail D-loop was detected upon inhibition of WRN branch migration after 15 min reaction time (data not shown). In summary, a protruding tail is not required for either WRN branch migration or exonuclease activities on a plasmid D-loop, and WRN can digest a 3′ ssDNA tail protruding from the D-loop.

**Figure 6:**
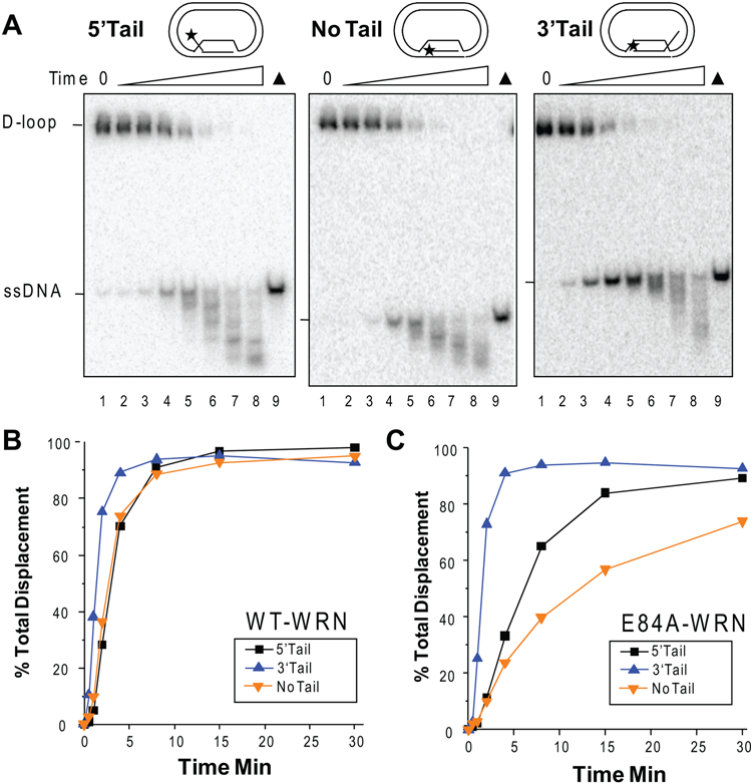
Kinetics of WRN activity on plasmid D-loops. Reactions contained 50 pM of either the 5′Tail, No Tail, or 3′Tail plasmid D-loops as indicated. The 5′ labeled end is indicated with a star. The substrate was incubated with 5 nM WRN (Panel A–B) or 5 nM E84A-WRN mutant (Panel C) for 0.5, 1, 2, 4, 8, 15, 30 min under standard reaction conditions. Reactions were run on 4–20% polyacrylamide native gels and visualized by phosphorimager analysis. ▴, heat denatured substrate. B. and C. The percent total displacement was calculated as described in Materials and Methods and plotted against time for reactions with wild type WRN (B.) or the E84A-WRN mutant (C.). 5′Tail D-loop, ▪ and black line; 3′Tail D-loop, ▴ and blue line; No Tail D-loop, ▾and yellow line. The representative phosphorimage scans for the E84A-WRN reactions are shown in Supplemental Fig. S3A.

To determine whether the presence of a protruding tail influences the kinetics of WRN D-loop disruption we conducted time course reactions. An increase in total strand displacement occurred as a function of time that began to plateau at about 4 min for the 3′Tail D-loop and 8 min for the 5′ and No Tail D-loops (Fig. 6A–B). The rate of total strand displacement was similar for the 5′ and No Tail D-loops, but was ∼2-fold higher for the 3′ Tail D-loop. Loss of the full length products in favor of extensively degraded products became apparent at the later time points, which most likely reflects additional digestion of the released ssDNA strands (Fig. 6A). To determine the influence of structure on the helicase branch migration activity alone, we examined the kinetics of D-loop disruption by the exonuclease-dead WRN mutant (E84A). The 3′ Tail D-loop was more rapidly disrupted by WRN branch migration activity than the 5′ and No Tail D-loops (Fig. 6C and Supplemental Fig. S3). Furthermore, the kinetics of strand displacement for the E84A-WRN mutant were similar to wild type WRN for the 3′Tail D-loop, but was decreased for the 5′Tail and No Tail D-loops, relative to wild type (Figs. 6B and C). This indicates that the WRN exonuclease activity contributes more to the rate of D-loop disruption for the substrates in which digestion proceeds into the duplex region (5′ and No Tail D-loop), than for the substrate in which duplex was not detectably shortened (3′ D-loop). The identical order of substrate preference was observed when wild type WRN exonuclease activity was inhibited by limiting Mg ^2+^ concentrations (Supplemental Fig. S3)

### WRN recognizes both junctions of the D-loop

**Figure 7:**
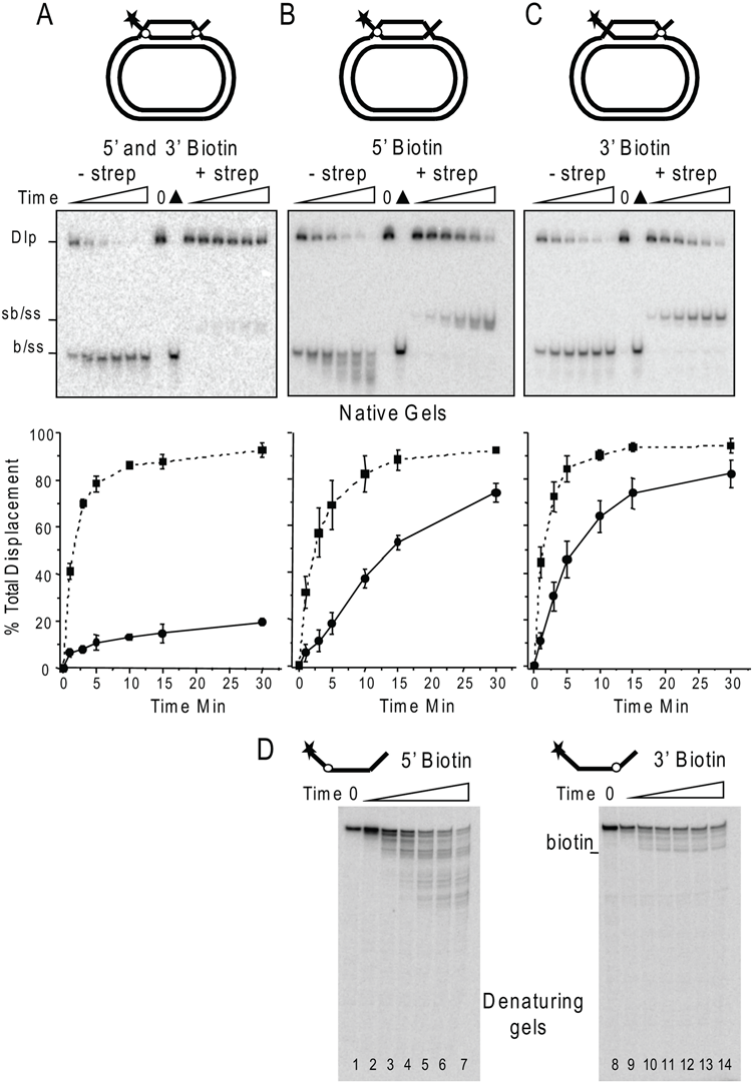
WRN recognized both ends of the invading strand duplex in a plasmid D-loop. A schematic is shown of the plasmid D-loops with a invading strand that forms a 60 bp duplex containing 18 nt protruding 5′ and 3′ ssDNA tails. A biotinylated nucleotide (circle) was positioned 2 nt from the duplex on the 3′ and 5′ tails (A), the 5′tail only (B) or the 3′tail only (C). Reactions contained 50 pM of plasmid D-loop that was pre-incubated for 10 min in reaction buffer alone or together with streptavidin as indicated. Then WRN (1.25 nM) was added and reaction aliquots were terminated at 0, 1, 3, 5, 10, 15 and 30 min. Reactions were run on 4–20% native polyacrylamide gels and visualized by phosphorimager analysis. ▴, heat denatured substrate. Dlp, D-loop; sb/ss, streptavidin-bound biotin ssDNA; b/ss biotin ssDNA. The percent total displacement was calculated as described in Materials and Methods and plotted against time. Biotin containing D-loops, ▪ and dotted line; Biotin+Strepavidin containing D-loops, • and solid line. Values represent the mean and standard deviation (SD) from at least three independent experiments. D. WRN exonuclease activity on ssDNA is inhibited by a biotin moiety. Reactions contained 50 pM of the 96-mer 5B Tail (lanes 1–7) or 3B Tail (lanes 8–14) oligonucleotides (Table 1). The substrates were incubated with 1.25 nM WRN and terminated after 0, 1, 3 5, 10, 15 and 30 reaction time under standard conditions. Reactions were run on 14% polyacrylamide denaturing gels.

**Figure 8:**
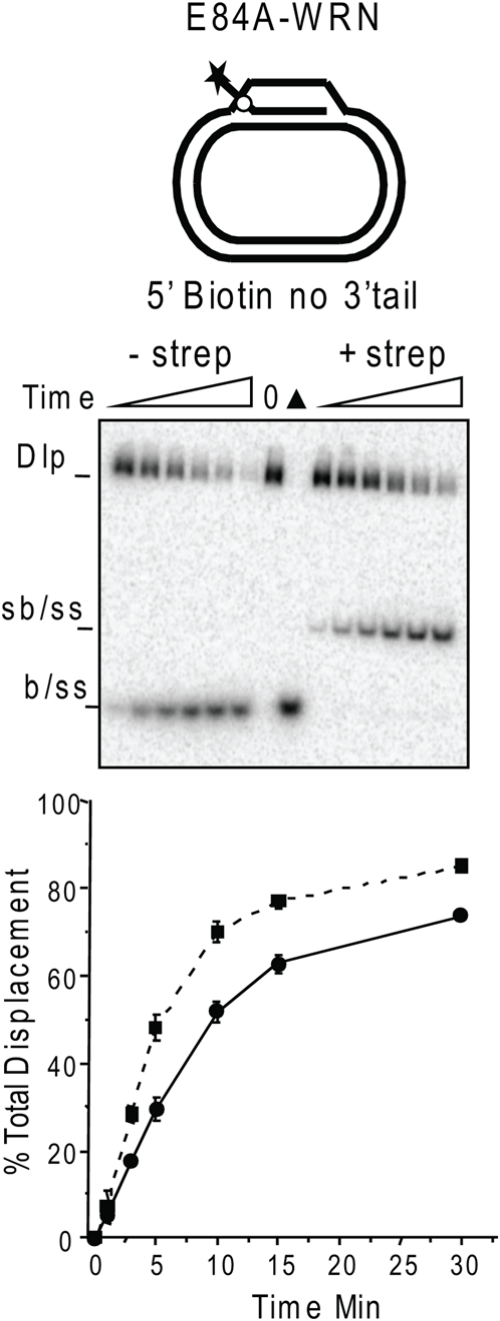
WRN disruption of D-loops with a 5′ blocked junction does not require a 3′tail or exonuclease activity. A schematic is shown of the plasmid D-loop with an invading strand that forms a 60 bp duplex containing a single 18 nt protruding 5′ ssDNA tail. A biotinylated nucleotide (circle) was positioned 2 nt from the duplex on the 5′ tail. Reactions contained 50 pM of plasmid D-loop that was pre-incubated for 10 min in reaction buffer alone or together with streptavidin as indicated. Then WRN (1.25 nM) was added and reaction aliquots were terminated at 0, 1, 3, 5, 10, 15 and 30 min. Reactions were run on 4–20% native polyacrylamide gels and visualized by phosphorimager analysis. ▴, heat denatured substrate. Dlp, D-loop; sb/ss, streptavidin-bound biotin ssDNA; b/ss biotin ssDNA. The percent total displacement was calculated as described in Materials and Methods and plotted against time. Biotin containing D-loops, ▪ and dotted line; Biotin+Strepavidin containing D-loops, • and solid line. Values represent the mean and standard deviation (SD) from two independent experiments.

Previous studies showed that WRN can bypass a streptavidin-biotin steric block on the 3′tail of a fork or flap substrate, indicating that WRN can recognize a fork-like junction directly to initiate unwinding. Based on this and other studies with oligomeric forks, D-loop and flap structures [36], [37], [49], we conjectured that WRN would load and initiate branch migration at the 5′ end of the invading strand duplex in the D-loop and translocate 3′ to 5′ along the plasmid strand. If this were true, the action of the WRN exonuclease at the opposite end (3′OH) would require the protein to simultaneously interact with both ends of the 84 bp duplex. Thus, we tested whether WRN D-loop disruption required WRN recognition of the 5′ or 3′end of the invading strand duplex to initiate displacement. We constructed plasmid D-loops containing an invading strand with both a 5′ and 3′ 18 nt ssDNA tail protruding from a 60 bp duplex (Fig. 7). A biotinylated nucleotide was incorporated in the 5′ and/or 3′ tail of the invading strand 2 nt away from the duplex region (junction). To impart a steric block at either the 5′ or 3′ junction or both, the D-loops were pre-incubated with streptavidin to bind the biotin groups. Free streptavidin protein does not inhibit WRN [49]. Importantly, the biotin moiety alone did not inhibit WRN branch migration activity as indicated by rapid disruption of the plasmid D-loops (Fig. 7). However, the WRN exonuclease cannot progress past the biotin (Fig. 7D) which explains the lack of detectable shortened products on the native gels for the D-loops with a biotin near the 3′end (Fig. 7 A and C). Blocking both the 5′ and 3′ junctions resulted in a dramatic reduction in D-loop disruption (Fig. 7A), indicating that the streptavidin-biotin complex effectively blocked WRN recognition of the junctions. In contrast, blocking only the 5′ junction slowed the rate of D-loop disruption by about 4.8-fold but did not prevent branch migration, suggesting that WRN can recognize the 3′ junction (Fig. 7B). Consistent with this, blocking only the 3′ junction also slowed the rate of D-loop disruption, in this case 2.7 fold (Fig. 7C). When either the 3′ or 5′ junction blocked, nearly the same maximal amount of D-loop disruption was eventually achieved as for the corresponding non-blocked D-loop (Fig. 7B–C). Furthermore, a WRN exonuclease-dead mutant can disrupt a D-loop containing a blocked 5′ junction that lacks a 3′ tail, indicating that WRN helicase branch migration from the accessible 3′junction does not require a 3′ tail for loading (Fig. 8). Importantly, the displaced strand exhibited slower mobility in the presence of streptavidin (Fig. 7–8). This confirms that streptavidin remained bound to DNA throughout the reactions, consistent with previous reports that WRN helicase does not dissociate the streptavidin from the DNA [49]. These data indicate that WRN protein can recognize both junction ends of the invading strand duplex, and can effectively disrupt D-loops containing a steric block at one junction but not when both junctions are blocked.

## Discussion

Increasing evidence supports an important role for WRN protein in mediating the proper dissociation of joint DNA molecules that arise in genomic and telomeric regions during homologous recombination and repair of breaks at collapsed replication forks. WRN was previously shown to displace and degrade the invading strand of static oligomeric-based D-loops with a 5′ tail or no tail, which were designed to mimic an important HR intermediate [36], [37]. However, WRN helicase was unable to efficiently unwind the full 33-bp invaded duplex in a telomeric D-loop without assistance from single strand binding proteins RPA and POT1 or the exonuclease to shorten the duplex [37], [38]. Here, we demonstrate that WRN helicase alone is capable of displacing a much longer telomeric and non-telomeric invading strand (84-bp) from plasmid based D-loop, presumably by promoting branch migration. However, D-loop processing is altered in the presence of WRN exonuclease activity which digests the invading strand both before and after release from the D-loop. We propose that mobile D-loops are particularly suitable HR structures for processing by WRN because they are substrates for both the helicase and exonuclease activities, unlike Holliday junction recombination intermediate. The increased activity of WRN on the more biologically relevant plasmid D-loops, compared to oligomeric D-loops, greatly strengthens the argument that WRN is capable of processing such structures at telomeric ends and during HR *in vivo*.

### WRN processing of static versus mobile D-loops

**Figure 9:**
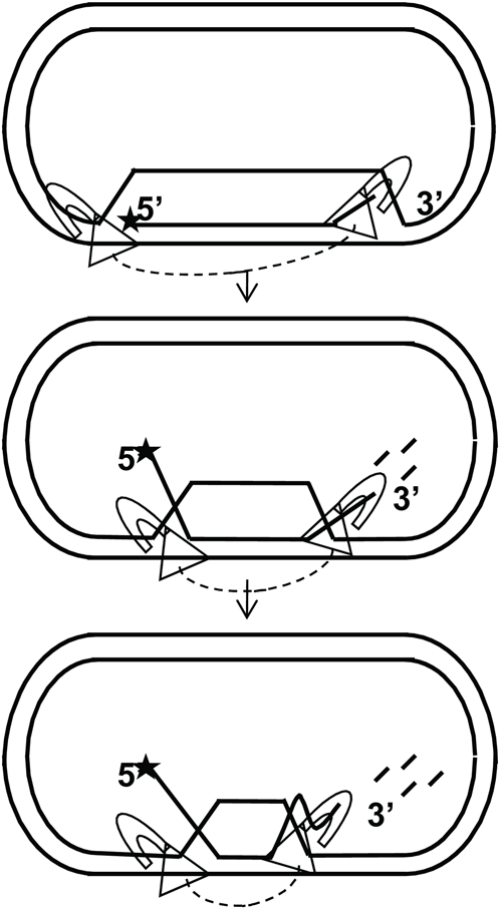
Bidirectional model for WRN branch migration and exonuclease activity on mobile D-loops. WRN protein with the helicase (triangle) and exonuclease (crescent) domains are depicted as separate monomers, but could also be linked in a higher oligomeric complex (denoted by a dotted line) such as a tetramer. The D-loops represent an intermediate in recombination, SDSA, and a structure at the telomeric end or ALT pathway. The D-loop depicted contains no pre-existing ssDNA tail although such tails may occur *in vivo*, such as a 3′ tail in a non-productive D-loop. WRN protein recognizes the 5′ duplex junction and helicase translocation along the plasmid strand in a 3′ to 5′ direction will promote branch migration. WRN also recognizes the 3′ duplex junction and may translocate along the invading strand 3′ to 5′ to promote branch migration, but can also digest the strand 3′ to 5′. In this model helicase driven branch migration proceeds in a bidirectional manner toward the middle causing shrinking of the displacement loop and subsequent release of the invading strand. Branch migration progresses more rapidly than the exonuclease which digests at one end and may cause a “loop out” of ssDNA (last structure). WRN exonuclease activity can also digest the strand after release depending on the length (not depicted here).

Our results indicate that WRN helicase and exonuclease process mobile D-loops and static D-loops by different mechanisms. We found that WRN helicase apparent processivity is increased on mobile D-loops, since WRN helicase alone can disrupt mobile D-loops with duplexes that are much longer (84 bp) (Figs. 2–3) than static D-loops that cannot be unwound (33 bp) [37]. We propose that by WRN helicase promoting branch migration through the mobile D-loop, the unpaired plasmid strands anneal and shrink the D-loop (Fig. 9). Thus, if WRN prematurely dissociates before the duplex is completely unwound, the partially unwound invading strand will not reanneal with the plasmid, allowing WRN to rapidly displace the remaining duplex upon rebinding the substrate. The loop cannot shrink in static D-loops since the “bubble” strands are not complementary and the partially unwound invading strand can reanneal if WRN prematurely dissociates. In agreement with our results, RECQ1 helicase also disrupts mobile D-loops by promoting branch migration, but is unable to unwind oligomeric D-loops of similar size [16]. Furthermore, BLM reactions with static D-loops produce intermediates that are not present in the mobile D-loop reactions [48]. Therefore the authors proposed that BLM may disrupt mobile D-loops, but not non-mobile D-loops, in a single step. Thus, WRN may displace mobile D-loops in a single binding step which could account for the increase in apparent processivity on mobile D-loops, compared to non-mobile D-loops which may require multiple binding steps for unwinding. Consistent with the increased apparent processivity of branch migration versus DNA unwinding, WRN was reported to promote migration through ∼2700 bp of duplex DNA in an α-structure recombination intermediate that resembles a Holliday junction [50]. In contrast, WRN unwinding of duplex DNA greater than ∼53 bp was found to require RPA [41](Supplemental Fig. S1).

### WRN loading on plasmid D-loops

Unlike RECQ1, WRN and BLM do not exhibit polarity of branch migration. RECQ1 branch migrates 3′ to 5′ and requires a 3′ tail [16], [19], however, BLM [48] and WRN (Fig. 5) do not require a tail for branch migration. Therefore, the loading site and the strand upon which WRN translocates are not obvious. WRN exonuclease activation at blunt ends of a fork occurs by protein loading at the forked end [42], [51], and so digestion only occurs on shorter forks (34-bp) and not longer ones (83-bp) (Fig. 1). WRN cannot load on blunt ends [47]. However, the D-loop contains a junction at both duplex ends and we found that accessibility of both junctions is required for optimal D-loop disruption by WRN, suggesting that WRN engages both junctions (Fig. 7). Thus, we propose a bidirectional model for WRN helicase driven branch migration of mobile D-loops (Fig. 9). In this scenario a WRN molecule engages the 5′ junction and translocates 3′ to 5′ along the plasmid strand, and another WRN molecule engages the 3′ junction and translocates 3′ to 5′ along the invading stand while simultaneously digesting the strand. Branch migration would proceed rapidly from both junctions, whereas the exonuclease activity occurs only at the available 3′OH end and progresses more slowly. Consistent with this, the products of branch migration through the 84 bp (full length strands) appeared earlier than the extensively digested products in the time course reactions (Fig. 6). This agrees with previous reports that the helicase progresses faster than the exonuclease activity [42]. In further support, we observed that blocking only one junction slowed the rate of D-loop disruption, but also altered the kinetic curve such that the reaction plateau was delayed (Fig. 7B–C). WRN eventually achieved near maximal D-loop disruption when one junction was blocked, but required more time than was needed for the corresponding unblocked D-loop (Fig. 7B–C). This suggests that WRN can achieve more rapid D-loop disruption when branch migration proceeds from both ends rather than one end. When both junctions were blocked, D-loop strand displacement was barely detectable even after 30 min (Fig. 7A). This suggests that initiation of branch migration was inhibited. However, we cannot distinguish whether separate WRN molecules engage the junction, or if different WRN monomers of a higher oligomeric complex engage the junctions. WRN was recently found to bind model replication forks and Holliday junctions [52] as a tetramer. Structural analysis and single molecule imaging will be key to deciphering these models.

Alternatively, WRN may only recognize and initiate branch migration from one junction in a unidirectional model. For example, the steric block at the 5′ junction might inhibit initiation whereas the steric block downstream at the 3′ junction might inhibit progression. We believe a biotin-streptavidin complex is more likely to inhibit WRN initiation rather than progression for several reasons. The downstream block is outside the duplex region and is also present on the opposite strand that WRN is presumed to track along in a unidirectional model. Bulky DNA adducts in duplex DNA strongly inhibit WRN when present on the translocating strand [53]. Furthermore, once the duplex is shortened to unstable lengths (∼12 bp) it will thermally melt [42], so a downstream block would be ∼14 nt away from WRN when strand displacement occurred. A biotin-streptavidin complex on oligomeric fork and flap substrates did not inhibit WRN unwinding unless the complex was placed at the junction [49]. Thus, we do not favor a unidirectional model.

### Comparison with other RecQ helicases

Caution should be applied when directly comparing our results with WRN to studies with other human RecQ helicases due to differences in the D-loop constructs examined (size and sequence) and reaction conditions. Nevertheless, WRN is at least as efficient as RECQ1 and BLM in disrupting various plasmid D-loops. In reactions for equivalent times and with similar amounts of 5′ tailed D-loops (∼50 pM), disruption of 50% of the D-loop is achieved with 7.4 nM BLM [48], compared to 0.5 nM WRN (Fig. 2) or ∼1 nM WRN if the exonuclease is inactivated (Fig. 3). RECQ1 preferentially unwinds 3′tailed D-loops, and disruption of approximately 50% of the substrate is achieved by 7.5 nM RECQ1 within ∼2.5 min [16], and by 5 nM WRN within ∼1.5 min even if the exonuclease is inactivated (Fig. 6). Thus, WRN is at least as efficient as BLM and RECQ1 but has the added feature of trimming the invading tails.

### Roles for WRN digestion of D-loop recombination intermediates

The activity of WRN exonuclease on plasmid D-loops affords WRN the ability to process these structures differently than other human RecQ helicases. Both the WRN exonuclease and helicase are required for the survival of recombinant progeny after the induction of Rad51-dependent HR intermediates to restore blocked or stalled DNA replication forks [22]–[24]. Our data indicate that both the WRN exonuclease and helicase are active on biologically relevant Rad51-dependent recombination intermediates. In addition to disrupting 3′ end invaded D-loops, we demonstrate for the first time that WRN can also disrupt 5′ end invaded D-loops (3′Tail D-loop) (Fig. 5). Rad51 promotes both 3′ and 5′ end invasion for D-loop production [54], [55], however, the latter is potentially toxic because it is unproductive. A 5′ end invaded D-loop cannot be extended by a polymerase to complete the next steps of HR repair of replication forks, SDSA, or the ALT pathways [6], [11]. While the role for the helicase in branch migration is clear, the role for the exonuclease has several possibilities. First, shortening of the invading strand results in fewer sites for Rad51 re-nucleation after strand release, which would inhibit repeated strand invasion events. Second, if the Rad51 mediated D-loop formation is incomplete such that the 3′ end is not fully paired with the template strand, it cannot be extended by a polymerase in the next steps of HR replication restart, SDSA or ALT pathways. WRN can rescue this unproductive intermediate by degrading the 3′ protruding ssDNA (Fig. 5).

### WRN activity on Telomeric D-loops

The decrease in WRN branch migration and exonuclease digestion of the telomeric sequence compared to the non-telomeric sequence have both biochemical and biological implications. Helicase processivity is influenced by base pair stability [56], therefore, differences could be related to duplex stability. However, we detected no obvious differences in thermal melting temperatures for the telomeric sequence compared to the non-telomeric sequence used in this study (Table 1) according to the HyTher web-based program [57]. The ssDNA TTAGGG repeats in the plasmid D-loop have the potential to form G-quadruplex DNA which could impede branch migration and would require resolution by the helicase [58]. The G-quadruplex DNA might also sequester WRN since WRN binds G4 DNA with high affinity [58]. The increased resistance of the telomeric D-loop to branch migration and digestion might also have implications for tighter regulation of processing at the telomeres *in vivo*. Inappropriate processing of telomeric ends by DNA repair proteins can have dire consequences for the cell and lead to senescence, apoptosis or genomic instability [33]. Untimely release of the telomeric t-loop/D-loop structure at the chromosome ends can activate a DNA damage response [59]. Therefore, resolution of telomeric D-loops might be more dependent on stimulation by telomeric proteins TRF2 and POT1, which regulate recombination at telomeric ends [33]. Consistent with this, POT1 and TRF2 interaction with WRN and were found to regulate WRN activities *in vitro* [37], [60].

In summary, we report that biologically relevant plasmid based D-loops with relatively long strand invasion duplex regions (84-bp) are substrates for both WRN branch migration and WRN exonuclease activity. WRN activity is not dependent on a protruding ssDNA tail, but the exonuclease is highly dependent on the free Mg^2+^ concentration. Our results offer a potential explanation for the cellular studies that indicate both WRN activities are required for dissociation of Rad51 dependent recombination intermediates to complete repair and suppress inappropriate recombination at stalled replication forks [25] and telomeric ends [29].

## Materials and Methods

### Proteins

Recombinant histidine-tagged WRN protein and the exonuclease-dead E84A mutant (X-WRN) were purified using a baculovirus/insect cell expression system and an AKTA Explorer FLPC (GE Healthcare, Piscataway, NJ) as previously described [60] except the HiLoad 16/60 Superdex 200 pg column was replaced with a HiTrap Phenyl HP column. Briefly, fractions containing WRN eluted off the HisTrap FF column were diluted to obtain final concentrations of 2 M NaCl in 20 mM phosphate buffer pH 7.4, 10% glycerol and 0.05% igepal CA-630. The eluent was loaded onto a HiTrap Phenyl HP column equilibrated with HIC buffer (20 mM phosphate buffer pH 7.4, 10% glycerol and 0.05% igepal CA-630) and washed once with HIC buffer containing 1.55 M NaCl prior to elution with 950 mM NaCl in HIC buffer. The eluted fractions containing WRN were loaded onto a HiTrap Desalt 5 ml column to exchange the buffer before loading onto a 1 ml Resource Q column for removal of remaining contaminants as described previously [60]. The concentration of active protein was determined by Bradford Assay (BioRad, Hercules, CA) and standard helicase reactions with a 16-bp forked duplex. Purity was determined by SDS-PAGE and Coomassie staining. All restriction enzymes were from New England Biolabs (Ipswich, MA). RecA protein was from USB Corporation (Cleveland, OH).

### DNA Substrates

All oligonucleotides used in this study (Table 1) were from Integrated DNA Technologies (Coralville, IA) and were purified by PAGE or HPLC by the manufacturer. Oligonucleotides were 5′ end-labeled with [γ-^32^P] ATP (3000 Ci/mmol) (Perkin Elmer, Waltham, MA) using T4 polynucleotide kinase (New England BioLabs, Ipswich, MA), according to the manufacturer's instructions. The plasimds used for making the various 84-bp non-telomeric and telomeric (Tel) D-loops were constructed by cloning the sequence (TTAGGG)10 (Tel10 plasmid) or the scrambled sequence 5′- ATC GGT ATT GGC TTA GCA CTG GCA ATC GGT CTT GCA CTG GCT ATT GGC TTA GGT ATC GCA (Non-tel 10 plasmid) between the bases 111 and 112 of the HSV-*tk* gene in the previously described pGTK4 plasmid [61]. The plasmids for constructing the 60-bp D-loops used in Figs. 7–8 contained the shorter insert sequence 5′-ATC GGT ATT GGC TTA GCA CTG GCA ATC GGT CTT GCA. The 6 kB plasmids were reduced to 3.6 kB to facilitate gel migration by removing 2363 bp through restriction with AccI followed by re-ligation. The plasmids were purified by two rounds of ethidium-bromide saturated CsCl equilibrium gradient ultracentrifugation (Loftstrand Labs, Gaithersburg, MD). The plasmid based D-loop substrates were constructed as described previously [62]. Briefly RecA (4 µM) was incubated with the invading strand oligonucleotide (3.6 µM nucleotide) for 5 min at 37°C, then the supercoiled plasmid (300 µM nucleotides) was added and incubated for 3 min more. The reactions were terminated by incubation with Proteinase K and SDS for 30 min as described previously [62]. The D-loop constructs were PAGE purified by using 4.5% (37.5∶1) polyacrylamide gels. After the bands were excised the gel slices were placed in dialysis D-tubes (Novagen, Madison, WI) and were subjected to two rounds of electroelution in 1×TBE for 2–4 hrs at 4°C and 120 V. The D-loops were concentrated and exchanged into storage buffer (10 mM Tris-HCl (pH 7.5), 10 mM MgCl_2_) using micron-30 devices (Amicon). Purification quality and yields were determined by analysis on 4–20% native polyacrylamide gels, followed by visualization and quantitation with a Typhoon phosphorimager and ImageQuant software (GE Healthcare, Piscataway, NJ). Restriction digest analysis of the invading strand in the plasmid D-loops was conducted in reactions (20 µl) containing 50 pM purified D-loops and 10 units of the restriction enzyme indicated in Fig. 1 for 4–5 hr at 37°C, according to the manufacturer's protocol. Products were analyzed on a 14% denaturing polyacrylamide gel.

The 83-bp long forked duplex was constructed by annealing 9 pmol of the 5′end labeled Fork-G oligonucleotide (Table 1) with 12 pmol of the Fork-C complementary strand in 50 µl with 50 mM LiCl at 95°C for 5 min, followed by cooling to room temperature. In order to achieve the final 83-bp length, the fork was restricted with HaeIII and then PAGE purified by running on 8% native polyacrylamide gel and using Qiaex II Gel Extraction Kit (Qiagen, Valencia, CA). Product purity and yield were determined by native PAGE and phosphorimager analysis.

### Helicase branch migration and exonuclease reactions

Reactions were performed in standard reaction buffer containing 40 mM Tris-HCl, pH 8.0, 4 mM MgCl_2_, 5 mM DTT, 100 µg/µl BSA, 14–28 ng/µl yeast tRNA, and 20 mM ATP [42] unless otherwise indicated. DNA substrate and protein concentrations were as indicated in the figure legends. The reactions were initiated by adding WRN protein and were incubated at 37°C for 15 minutes, unless otherwise indicated. For reactions with D-loops containing a biotinylated nucleotide the substrate was pre-incubated at 37°C for 10 minutes in buffer alone or with 1.5 nM streptavidin prior to WRN addition. For analysis of radiolabeled molecules on 4–20% native polyacrylamide gels, the reactions (10 µL) were stopped with 5 µL of 3× stop dye supplemented with 10 µg/mL proteinase K [42] and de-proteinized for 15 min at 37°C. For analysis of the radio-labeled molecules on 14% denaturing gels, the reactions were terminated with an equal volume of formamide stop dye [42]. The helicase reactions with the 83-bp forked duplex were terminated in 3× stop dye with a 10-fold molar excess of cold competitor oligonucleotide to prevent reannealing of the unwound products. After drying the gels, the reactions were visualized using a Typhoon phosphorimager and quantified using ImageQuant software (GE Healthcare, Piscataway, NJ).

For quantitation of strand displacement the percent of displaced products (full length and shortened) were calculated as a function of the total radioactivity in the reaction lane [37]. All values were corrected for background in the no enzyme control and heat denatured substrate lanes.

## Supporting Information

Figure S1 WRN helicase cannot completely unwind an 83 bp telomeric forked duplex. Reactions contained 50 pM of the 84-bp forked duplex containing ten telomeric repeats (thick black line) flanked by 15 and 8 bp of unique sequence. The substrate was incubated with WRN concentrations increasing by doubling steps from 0.039 to 5 nM for 15 min under standard reaction conditions. Reactions were run on an 8% native gel.
(1.17 MB EPS)

Figure S2 WRN exonuclease digests free 120 nt single strands. Reactions contained 50 pM of the 5′Tail non-telomeric oligonucleotide (panel A, lanes 1–7; panel B, lanes 1–4) or the telomeric (5′Tail Tel) oligonucleotide (panel A, lanes 8–14; panel B, lanes 5–8) (see Table 1). The substrate was incubated with increasing WRN concentrations (0, 0.15, 0.31, 0.62, 1.2, 2.5 or 5 nM) (panel A) or E84A-WRN mutant (0, 1.2, 2.5, or 5 nM) (panel B) for 15 min under standard reaction conditions. Reactions were run on a 14% denaturing polyacrylamide gel.
(3.57 MB EPS)

Figure S3 Kinetics of WRN branch migration activity on plasmid D-loops. Reactions contained 50 pM of either the 5′Tail, No Tail, or 3′ Tail plasmid D-loops as indicated. The 5′ labeled end is indicated with a star. The substrate was incubated with 5 nM E84A-WRN under standard reaction conditions (Panel A) or wild type WRN (Panel B) under modified conditions in which Mg^2+^ was reduced to 1 mM. Aliquots were removed at 0, 0.5, 1, 2, 4, 8, 15, 30 min and terminated. Reactions were run on 4–20% polyacrylamide native gels and visualized by phosphorimager analysis. Δ, heat denatured substrate. The quantification of the reactions in Panel A is shown in Fig. 6C. B. The percent total displacement was calculated as described in Materials and Methods and plotted against time for reactions with wild type WRN and limiting Mg^2+^. 5′Tail D-loop, square and black line; 3′ Tail D-loop, triangle and blue line; No Tail D-loop, inverted triangle and yellow line.
(2.53 MB EPS)
